# [η^5^-2,3-Bis(trimethylsilyl)-2,3-dicarba-*nido*-hexaborane(2−)]chlorido(*N*,*N*,*N*′,*N*′-tetramethylethylenediamine)dysprosium(III)

**DOI:** 10.1107/S1600536808013615

**Published:** 2008-05-10

**Authors:** Chong Zheng, Graciela Canseco-Melchor, John A. Maguire, Narayan S. Hosmane

**Affiliations:** a Department of Chemistry and Biochemistry, Northern Illinois University, DeKalb, IL 60115, USA; b Department of Chemistry, Southern Methodist University, Dallas, TX 75275, USA

## Abstract

The structure of the title compound, [Dy(C_8_H_22_B_4_Si_2_)Cl(C_6_H_16_N_2_)], reveals that a center of symmetry exists within the dimeric half-sandwich units. Within each half-sandwich, the Dy^III^ ion is coordinated by the five-membered ring of the carborane, tetramethylethyl­enediamine and the chloride ion.

## Related literature

For related literature, see: Bazan *et al.* (1993 ▶); Tomlinson *et al.* (2005 ▶); Wang *et al.* (2006 ▶).
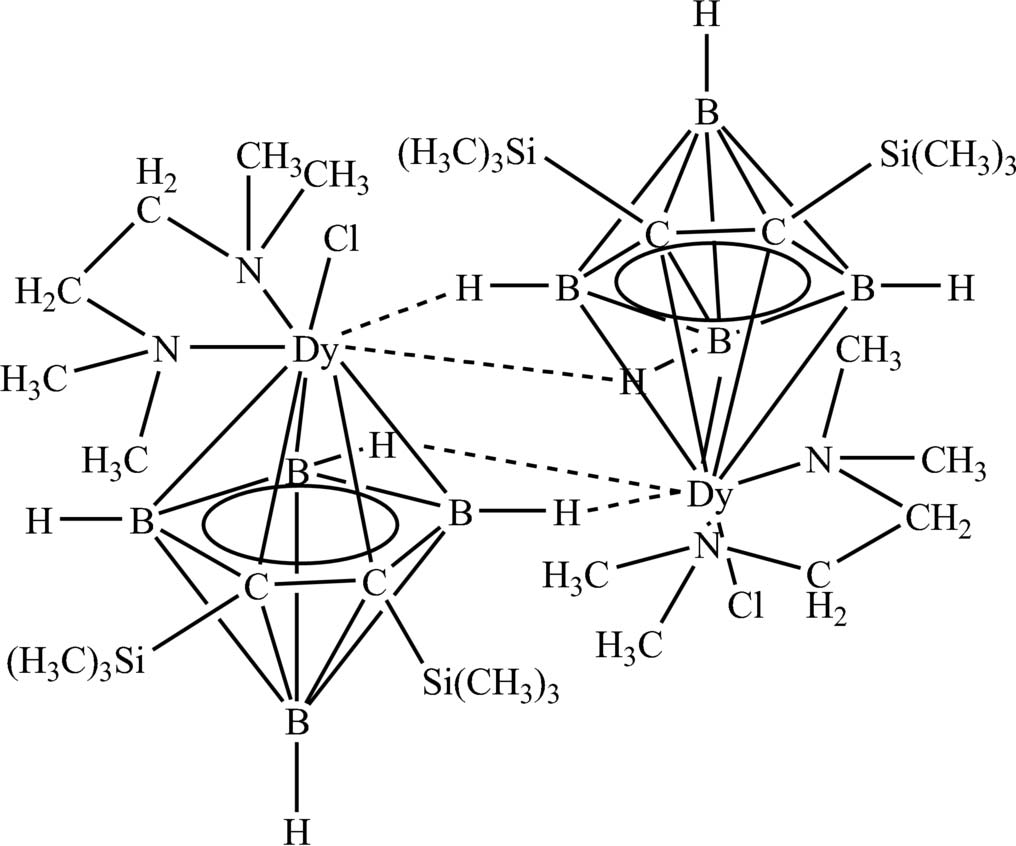


## Experimental

### 

#### Crystal data

[Dy(C_8_H_22_B_4_Si_2_)Cl(C_6_H_16_N_2_)]
*M* *_r_* = 531.84
Monoclinic, 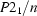

*a* = 11.4467 (8) Å
*b* = 14.862 (1) Å
*c* = 13.8615 (9) Å
β = 92.770 (1)°
*V* = 2355.4 (3) Å^3^
*Z* = 4
Mo *K*α radiation
μ = 3.39 mm^−1^
*T* = 293 (2) K
0.80 × 0.30 × 0.30 mm

#### Data collection

Bruker SMART CCD PLATFORM diffractometer
Absorption correction: multi-scan *SADABS* (Sheldrick, 2006 ▶) *T*_min_ = 0.257, *T*_max_ = 0.362
17271 measured reflections
4151 independent reflections
4142 reflections with *I* > 2σ(*I*)
*R*_int_ = 0.019

#### Refinement

*R*[*F* ^2^ > 2σ(*F* ^2^)] = 0.024
*wR*(*F* ^2^) = 0.050
*S* = 1.44
4151 reflections
231 parameters
H atoms treated by a mixture of independent and constrained refinement
Δρ_max_ = 0.64 e Å^−3^
Δρ_min_ = −0.61 e Å^−3^

### 

Data collection: *SMART* (Bruker, 2003 ▶); cell refinement: *SMART* and *SAINT* (Bruker, 2003 ▶); data reduction: *SAINT* (Bruker, 2003 ▶); program(s) used to solve structure: *SIR97* (Altomare *et al.*, 1999 ▶); program(s) used to refine structure: *SHELXTL* (Sheldrick, 2008 ▶); molecular graphics: *SHELXTL*; software used to prepare material for publication: *SHELXTL*.

## Supplementary Material

Crystal structure: contains datablocks global, I. DOI: 10.1107/S1600536808013615/si2081sup1.cif

Structure factors: contains datablocks I. DOI: 10.1107/S1600536808013615/si2081Isup2.hkl

Additional supplementary materials:  crystallographic information; 3D view; checkCIF report

## Figures and Tables

**Fig. 1.**
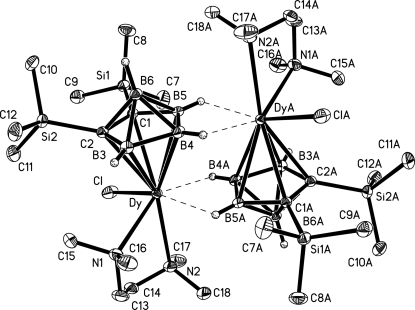
Thermal ellipsoid drawing of the title compound shown at the 30% probability level. Atoms of the symmetry related moiety of the dimer complex are indicated "A" (symmetry code 1 - x, 2 - y, 1 - z).

**Fig. 2.**
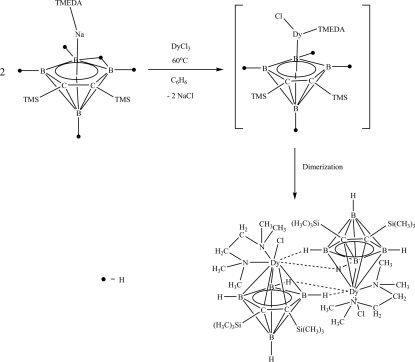
Reaction scheme

## supplementary crystallographic information

### Comment

The lanthanacarboranes (LnC_2_B_4_) cage systems are of interest in that their structural chemistry depends on the number and nature of the metal ligands other than the particular carborane. For example, the reaction of the tetrahydrofuran (THF) solvated dilithium compounds of B_4_C_8_H_22_Si_2_^2-^ with LnCl_3_ in 2:1 molar ratios produced exclusively a trinuclear clusters (Tomlinson *et al.* 2005, Wang *et al.* 2006, and literature cited therein). The methoxide and oxide ions were thought to be the result of a degradation of the THF molecules. This is consistent with the observation that the reaction of the TMEDA lithiacarboranes with LnCl_3_ in 2:1 molar ratios gave the expected full sandwich lanthanacarboranes. This tendency for TMEDA to disrupt complex aggregation and alter the course of a metalation reactions was also found in the d-block metals. The reaction of the THF solvated dilithium salts of B_4_C_8_H_22_Si_2_^2-^ with either CoCl_2_ or NiCl_2_ induced a metal disproportion reaction yielding *commo*-complexes and the respective metals. On the other hand, similar reactions with the TMEDA-solvated carborane dianions produced the corresponding *closo*-compound (Tomlinson *et al.* 2005). The reaction of a *nido*-compound with a number of lanthanide halides in 2:1 carborane-to-lanthanide molar ratios produced only the corresponding dimers (Wang *et al.* 2006). The presence of the reactive chlorides made these compounds potentially useful precursors in the syntheses of other lanthanacarboranes. Since dimer formation is thought to inhibit the reactions of the metallacatboranes (Bazan *et al.* 1993), the synthesis of a monomeric dysprosacarborane was attempted by the reaction of DyCl_3_ and the *nido*-compound, giving the title compound. The structure of the title compound, shown in Figure 1, is somewhat surprising in that it still remains a dimer. The structure consists of two half-sandwich units bridged at the B4, B5 positions by two pairs of B—H—Dy bonds. The average coordination bond distances (Å) are: Dy—Cl 2.5851 (9), Dy—N1 2.598 (3), Dy—N2 2.698 (3), Dy—B4,B5 2.728 (4), 2.692 (4), Dy-av(C1, C2, B3, B4, B5) 2.720 and the corresponding bond angles (°) are Cl—Dy—N1 95.98 (7), Cl—Dy—B5 143.74 (8), N(1)—Dy—N(2) 68.3 (1), B(5)—Dy—B(4) 35.7 (1). The Dy-av(C_2_B_3_) distance of 2.712Å and the Dy—Cl distance of 2.5851 (9) Å in the structure are similar to the values of 2.717 Å and 2.5757 (14) Å, respectively, found in anther Dy compound (Wang *et al.* 2006). This is one of the few instances where the substitution of THF by TMEDA molecules does not change either the cluster or intermolecular geometries.

### Experimental

The reaction of DyCl_3_ with the TMEDA solvated monosodium *nido*- compound, *nido*-1-Na(TMEDA)2–2,3-(SiMe_3_)_2_-2,3-C_2_B_4_H_4_, in a 1:2 molar ratio in dry benzene at 60° C produced the corresponding half sandwich carborane in 88% yield.

### Refinement

The hydrogen atoms on carbon atoms were refined using the riding model in *SHELXL* with the *U*_iso_ equal to 1.5 times of that of the preceding carbon atoms for the methyl groups and 1.3 times for the rings. The C—H distances are equal to 0.97 and 0.96 Å for the CH_2_ and CH_3_ groups, respectively. The hydrogen atoms attached to boron atoms were located using the difference map, those of the carborane ring were refined using the riding model with B—H distances 0.93 Å. The hydrogen atom attached to B6 was refined with an isotropic displacement parameter with B—H distance 0.96 Å. The *U*_iso_(H) = 1.2 times *U*_eq_(B3,B4,B5).

### Crystal data

**Table:**

[Dy(C_8_H_22_B_4_Si_2_)Cl(C_6_H_16_N_2_)]	*F*_000_ = 1068
*M**_r_* = 531.84	*D*_x_ = 1.500 Mg m^−^^3^
Monoclinic, *P*2_1_/*n*	Mo *K*α radiation λ = 0.71073 Å
Hall symbol: -P 2yn	Cell parameters from 562 reflections
*a* = 11.4467 (8) Å	θ = −14–14º
*b* = 14.8624 (10) Å	µ = 3.39 mm^−^^1^
*c* = 13.8615 (9) Å	*T* = 293 (2) K
β = 92.7700 (10)º	Column, colorless
*V* = 2355.4 (3) Å^3^	0.80 × 0.30 × 0.30 mm
*Z* = 4	

### Data collection

**Table:**

Bruker SMART CCD PLATFORM diffractometer	4151 independent reflections
Radiation source: fine-focus sealed tube	4142 reflections with *I* > 2σ(*I*)
Monochromator: graphite	*R*_int_ = 0.019
Detector resolution: 0 pixels mm^-1^	θ_max_ = 25.0º
*T* = 293(2) K	θ_min_ = 2.0º
ω scans	*h* = −13→13
Absorption correction: multi-scanSADABS (Sheldrick, 2006)	*k* = −17→17
*T*_min_ = 0.257, *T*_max_ = 0.362	*l* = −16→16
17271 measured reflections	

### Refinement

**Table:**

Refinement on *F*^2^	Secondary atom site location: difference Fourier map
Least-squares matrix: full	Hydrogen site location: inferred from neighbouring sites
*R*[*F*^2^ > 2σ(*F*^2^)] = 0.024	H atoms treated by a mixture of independent and constrained refinement
*wR*(*F*^2^) = 0.050	*w* = 1/[σ^2^(*F*_o_^2^) + 3.9928*P*] where *P* = (*F*_o_^2^ + 2*F*_c_^2^)/3
*S* = 1.44	(Δ/σ)_max_ = 0.013
4151 reflections	Δρ_max_ = 0.64 e Å^−^^3^
231 parameters	Δρ_min_ = −0.61 e Å^−^^3^
Primary atom site location: structure-invariant direct methods	Extinction correction: none

### Special details

**Table:**

Geometry. All e.s.d.'s (except the e.s.d. in the dihedral angle between two l.s. planes) are estimated using the full covariance matrix. The cell e.s.d.'s are taken into account individually in the estimation of e.s.d.'s in distances, angles and torsion angles; correlations between e.s.d.'s in cell parameters are only used when they are defined by crystal symmetry. An approximate (isotropic) treatment of cell e.s.d.'s is used for estimating e.s.d.'s involving l.s. planes.
Refinement. Refinement of *F*^2^ against ALL reflections. The weighted *R*-factor *wR* and goodness of fit *S* are based on *F*^2^, conventional *R*-factors *R* are based on *F*, with *F* set to zero for negative *F*^2^. The threshold expression of *F*^2^ > σ(*F*^2^) is used only for calculating *R*-factors(gt) *etc*. and is not relevant to the choice of reflections for refinement. *R*-factors based on *F*^2^ are statistically about twice as large as those based on *F*, and *R*- factors based on ALL data will be even larger.

### Fractional atomic coordinates and isotropic or equivalent isotropic displacement parameters (Å^2^)

**Table:**

	*x*	*y*	*z*	*U*_iso_*/*U*_eq_	
Dy	0.584689 (12)	0.954047 (10)	0.626856 (11)	0.02053 (6)	
Cl	0.65147 (8)	0.79787 (6)	0.69040 (8)	0.0406 (2)	
Si1	0.37951 (8)	0.73248 (6)	0.55959 (7)	0.0268 (2)	
Si2	0.31576 (8)	0.84186 (6)	0.80092 (7)	0.0269 (2)	
C1	0.3894 (3)	0.8543 (2)	0.5966 (2)	0.0205 (6)	
C2	0.3757 (3)	0.8993 (2)	0.6921 (2)	0.0221 (7)	
B3	0.3682 (3)	1.0040 (3)	0.6794 (3)	0.0249 (8)	
H3	0.3640	1.0465	0.7285	0.030*	
B4	0.3693 (3)	1.0245 (2)	0.5630 (3)	0.0233 (8)	
H4	0.3622	1.0799	0.5320	0.028*	
B5	0.3862 (3)	0.9239 (2)	0.5134 (3)	0.0217 (7)	
H5	0.3926	0.9117	0.4481	0.026*	
B6	0.2804 (3)	0.9338 (3)	0.6029 (3)	0.0239 (8)	
C7	0.4863 (4)	0.7136 (3)	0.4645 (3)	0.0501 (11)	
H7A	0.4791	0.6531	0.4410	0.075*	
H7B	0.5642	0.7232	0.4914	0.075*	
H7C	0.4707	0.7550	0.4123	0.075*	
C8	0.2302 (4)	0.7083 (3)	0.5048 (3)	0.0472 (11)	
H8A	0.1723	0.7344	0.5441	0.071*	
H8B	0.2185	0.6444	0.5012	0.071*	
H8C	0.2230	0.7336	0.4411	0.071*	
C9	0.4155 (3)	0.6475 (2)	0.6554 (3)	0.0380 (9)	
H9A	0.3536	0.6453	0.6996	0.057*	
H9B	0.4873	0.6639	0.6896	0.057*	
H9C	0.4242	0.5894	0.6262	0.057*	
C10	0.1876 (3)	0.7697 (3)	0.7643 (3)	0.0372 (9)	
H10A	0.1693	0.7311	0.8170	0.056*	
H10B	0.2062	0.7338	0.7095	0.056*	
H10C	0.1214	0.8072	0.7474	0.056*	
C11	0.4250 (4)	0.7748 (3)	0.8752 (3)	0.0450 (10)	
H11A	0.4679	0.8139	0.9191	0.068*	
H11B	0.4780	0.7458	0.8335	0.068*	
H11C	0.3850	0.7300	0.9112	0.068*	
C12	0.2567 (4)	0.9287 (3)	0.8824 (3)	0.0436 (10)	
H12A	0.3179	0.9698	0.9023	0.065*	
H12B	0.2270	0.9000	0.9382	0.065*	
H12C	0.1947	0.9611	0.8487	0.065*	
N1	0.6388 (3)	1.0410 (2)	0.7853 (2)	0.0323 (7)	
C13	0.7672 (3)	1.0466 (3)	0.8066 (3)	0.0430 (10)	
H13A	0.7826	1.0429	0.8760	0.052*	
H13B	0.7950	1.1046	0.7854	0.052*	
C14	0.8344 (3)	0.9737 (3)	0.7587 (3)	0.0367 (9)	
H14A	0.9169	0.9798	0.7768	0.044*	
H14B	0.8086	0.9155	0.7812	0.044*	
N2	0.8178 (3)	0.9778 (2)	0.6525 (2)	0.0345 (7)	
C15	0.5868 (4)	0.9852 (3)	0.8603 (3)	0.0442 (10)	
H15A	0.6170	0.9250	0.8572	0.066*	
H15B	0.5034	0.9840	0.8496	0.066*	
H15C	0.6064	1.0102	0.9229	0.066*	
C16	0.5897 (4)	1.1327 (3)	0.7910 (3)	0.0464 (10)	
H16A	0.6069	1.1570	0.8543	0.070*	
H16B	0.5064	1.1303	0.7788	0.070*	
H16C	0.6237	1.1704	0.7436	0.070*	
C17	0.8846 (3)	0.9040 (3)	0.6097 (4)	0.0554 (12)	
H17A	0.9663	0.9113	0.6268	0.083*	
H17B	0.8727	0.9054	0.5407	0.083*	
H17C	0.8581	0.8474	0.6338	0.083*	
C18	0.8693 (4)	1.0633 (3)	0.6189 (3)	0.0517 (12)	
H18A	0.9477	1.0692	0.6460	0.078*	
H18B	0.8230	1.1130	0.6392	0.078*	
H18C	0.8706	1.0628	0.5497	0.078*	
H6	0.191 (3)	0.916 (2)	0.600 (3)	0.029 (9)*	

### Atomic displacement parameters (Å^2^)

**Table:**

	*U*^11^	*U*^22^	*U*^33^	*U*^12^	*U*^13^	*U*^23^
Dy	0.01951 (8)	0.01862 (9)	0.02317 (9)	−0.00146 (6)	−0.00200 (6)	0.00244 (6)
Cl	0.0311 (5)	0.0249 (4)	0.0647 (6)	0.0002 (4)	−0.0081 (4)	0.0114 (4)
Si1	0.0314 (5)	0.0189 (5)	0.0301 (5)	−0.0025 (4)	0.0010 (4)	−0.0016 (4)
Si2	0.0303 (5)	0.0268 (5)	0.0238 (5)	−0.0015 (4)	0.0049 (4)	0.0036 (4)
C1	0.0178 (15)	0.0198 (16)	0.0238 (16)	−0.0006 (12)	0.0005 (12)	0.0010 (13)
C2	0.0196 (16)	0.0224 (16)	0.0242 (17)	−0.0003 (13)	0.0008 (13)	0.0005 (13)
B3	0.0251 (19)	0.0226 (19)	0.027 (2)	0.0016 (15)	0.0014 (15)	−0.0026 (15)
B4	0.0221 (18)	0.0163 (18)	0.031 (2)	0.0009 (14)	0.0003 (15)	0.0034 (15)
B5	0.0184 (17)	0.0230 (19)	0.0237 (18)	−0.0028 (14)	−0.0002 (14)	−0.0003 (15)
B6	0.0207 (18)	0.0231 (19)	0.028 (2)	0.0002 (15)	0.0010 (15)	0.0021 (15)
C7	0.067 (3)	0.036 (2)	0.049 (3)	0.008 (2)	0.022 (2)	−0.0054 (19)
C8	0.050 (3)	0.036 (2)	0.054 (3)	−0.0117 (19)	−0.019 (2)	−0.0039 (19)
C9	0.039 (2)	0.0247 (19)	0.049 (2)	−0.0006 (16)	−0.0026 (18)	0.0039 (17)
C10	0.033 (2)	0.040 (2)	0.040 (2)	−0.0074 (17)	0.0114 (17)	0.0021 (17)
C11	0.048 (2)	0.045 (2)	0.041 (2)	0.0010 (19)	−0.0038 (19)	0.0168 (19)
C12	0.053 (3)	0.042 (2)	0.036 (2)	−0.0025 (19)	0.0161 (19)	−0.0034 (18)
N1	0.0360 (17)	0.0301 (16)	0.0303 (16)	−0.0061 (13)	−0.0058 (13)	−0.0029 (13)
C13	0.037 (2)	0.047 (2)	0.044 (2)	−0.0102 (18)	−0.0129 (18)	−0.0051 (19)
C14	0.0300 (19)	0.039 (2)	0.039 (2)	−0.0056 (16)	−0.0141 (16)	0.0089 (17)
N2	0.0266 (15)	0.0383 (18)	0.0377 (17)	−0.0059 (13)	−0.0058 (13)	0.0031 (14)
C15	0.052 (2)	0.053 (3)	0.027 (2)	−0.011 (2)	−0.0055 (18)	0.0033 (18)
C16	0.053 (3)	0.034 (2)	0.052 (3)	−0.0002 (19)	−0.005 (2)	−0.0141 (19)
C17	0.025 (2)	0.068 (3)	0.073 (3)	−0.001 (2)	0.002 (2)	−0.016 (3)
C18	0.037 (2)	0.068 (3)	0.049 (3)	−0.023 (2)	−0.0137 (19)	0.020 (2)

### Geometric parameters (Å, °)

**Table:**

Dy—Cl	2.5851 (9)	C9—H9B	0.9600
Dy—N1	2.598 (3)	C9—H9C	0.9600
Dy—B5^i^	2.692 (4)	C10—H10A	0.9600
Dy—N2	2.698 (3)	C10—H10B	0.9600
Dy—C1	2.698 (3)	C10—H10C	0.9600
Dy—C2	2.723 (3)	C11—H11A	0.9600
Dy—B4^i^	2.728 (4)	C11—H11B	0.9600
Si1—C7	1.861 (4)	C11—H11C	0.9600
Si1—C9	1.865 (4)	C12—H12A	0.9600
Si1—C8	1.872 (4)	C12—H12B	0.9600
Si1—C1	1.884 (3)	C12—H12C	0.9600
Si2—C12	1.864 (4)	N1—C15	1.477 (5)
Si2—C10	1.867 (4)	N1—C16	1.477 (5)
Si2—C11	1.869 (4)	N1—C13	1.487 (5)
Si2—C2	1.890 (3)	C13—C14	1.502 (6)
C1—C2	1.498 (4)	C13—H13A	0.9700
C1—B5	1.548 (5)	C13—H13B	0.9700
C1—B6	1.724 (5)	C14—N2	1.477 (5)
C2—B3	1.568 (5)	C14—H14A	0.9700
C2—B6	1.689 (5)	C14—H14B	0.9700
B3—B4	1.642 (5)	N2—C17	1.478 (5)
B3—H3	0.9300	N2—C18	1.485 (5)
B4—B5	1.661 (5)	C15—H15A	0.9600
B4—Dy^i^	2.728 (4)	C15—H15B	0.9600
B4—H4	0.9300	C15—H15C	0.9600
B5—Dy^i^	2.692 (4)	C16—H16A	0.9600
B5—H5	0.9300	C16—H16B	0.9600
B6—H6	1.06 (4)	C16—H16C	0.9600
C7—H7A	0.9600	C17—H17A	0.9600
C7—H7B	0.9600	C17—H17B	0.9600
C7—H7C	0.9600	C17—H17C	0.9600
C8—H8A	0.9600	C18—H18A	0.9600
C8—H8B	0.9600	C18—H18B	0.9600
C8—H8C	0.9600	C18—H18C	0.9600
C9—H9A	0.9600		
			
Cl—Dy—N1	95.98 (7)	Si1—C8—H8B	109.5
Cl—Dy—B5^i^	143.74 (8)	H8A—C8—H8B	109.5
N1—Dy—B5^i^	104.06 (10)	Si1—C8—H8C	109.5
Cl—Dy—N2	78.36 (7)	H8A—C8—H8C	109.5
N1—Dy—N2	68.26 (10)	H8B—C8—H8C	109.5
B5^i^—Dy—N2	81.43 (10)	Si1—C9—H9A	109.5
Cl—Dy—C1	77.81 (7)	Si1—C9—H9B	109.5
N1—Dy—C1	124.51 (10)	H9A—C9—H9B	109.5
B5^i^—Dy—C1	112.86 (10)	Si1—C9—H9C	109.5
N2—Dy—C1	154.04 (9)	H9A—C9—H9C	109.5
Cl—Dy—C2	82.45 (7)	H9B—C9—H9C	109.5
N1—Dy—C2	92.59 (10)	Si2—C10—H10A	109.5
B5^i^—Dy—C2	125.64 (10)	Si2—C10—H10B	109.5
N2—Dy—C2	151.01 (9)	H10A—C10—H10B	109.5
C1—Dy—C2	32.08 (9)	Si2—C10—H10C	109.5
Cl—Dy—B4^i^	111.47 (8)	H10A—C10—H10C	109.5
N1—Dy—B4^i^	135.13 (10)	H10B—C10—H10C	109.5
B5^i^—Dy—B4^i^	35.69 (11)	Si2—C11—H11A	109.5
N2—Dy—B4^i^	82.89 (10)	Si2—C11—H11B	109.5
C1—Dy—B4^i^	96.39 (10)	H11A—C11—H11B	109.5
C2—Dy—B4^i^	124.68 (10)	Si2—C11—H11C	109.5
C7—Si1—C9	105.8 (2)	H11A—C11—H11C	109.5
C7—Si1—C8	107.6 (2)	H11B—C11—H11C	109.5
C9—Si1—C8	108.95 (19)	Si2—C12—H12A	109.5
C7—Si1—C1	107.74 (17)	Si2—C12—H12B	109.5
C9—Si1—C1	116.69 (16)	H12A—C12—H12B	109.5
C8—Si1—C1	109.69 (17)	Si2—C12—H12C	109.5
C12—Si2—C10	105.03 (19)	H12A—C12—H12C	109.5
C12—Si2—C11	106.8 (2)	H12B—C12—H12C	109.5
C10—Si2—C11	109.84 (19)	C15—N1—C16	108.3 (3)
C12—Si2—C2	109.07 (17)	C15—N1—C13	108.6 (3)
C10—Si2—C2	110.65 (16)	C16—N1—C13	108.3 (3)
C11—Si2—C2	114.95 (17)	C15—N1—Dy	103.2 (2)
C2—C1—B5	111.1 (3)	C16—N1—Dy	115.3 (2)
C2—C1—B6	62.8 (2)	C13—N1—Dy	112.9 (2)
B5—C1—B6	65.7 (2)	N1—C13—C14	113.2 (3)
C2—C1—Si1	131.5 (2)	N1—C13—H13A	108.9
B5—C1—Si1	116.1 (2)	C14—C13—H13A	108.9
B6—C1—Si1	129.7 (2)	N1—C13—H13B	108.9
C2—C1—Dy	74.86 (17)	C14—C13—H13B	108.9
B5—C1—Dy	74.80 (17)	H13A—C13—H13B	107.7
B6—C1—Dy	102.23 (18)	N2—C14—C13	111.5 (3)
Si1—C1—Dy	127.49 (14)	N2—C14—H14A	109.3
C1—C2—B3	110.6 (3)	C13—C14—H14A	109.3
C1—C2—B6	65.2 (2)	N2—C14—H14B	109.3
B3—C2—B6	65.6 (2)	C13—C14—H14B	109.3
C1—C2—Si2	124.1 (2)	H14A—C14—H14B	108.0
B3—C2—Si2	121.2 (2)	C14—N2—C17	109.0 (3)
B6—C2—Si2	118.4 (2)	C14—N2—C18	108.3 (3)
C1—C2—Dy	73.05 (17)	C17—N2—C18	106.7 (3)
B3—C2—Dy	73.14 (18)	C14—N2—Dy	101.8 (2)
B6—C2—Dy	102.27 (19)	C17—N2—Dy	112.2 (2)
Si2—C2—Dy	139.32 (15)	C18—N2—Dy	118.4 (2)
C2—B3—B4	106.9 (3)	N1—C15—H15A	109.5
C2—B3—H3	126.5	N1—C15—H15B	109.5
B4—B3—H3	126.5	H15A—C15—H15B	109.5
B3—B4—B5	104.3 (3)	N1—C15—H15C	109.5
B3—B4—Dy^i^	168.7 (2)	H15A—C15—H15C	109.5
B5—B4—Dy^i^	70.97 (18)	H15B—C15—H15C	109.5
B3—B4—H4	127.9	N1—C16—H16A	109.5
B5—B4—H4	127.9	N1—C16—H16B	109.5
Dy^i^—B4—H4	57.8	H16A—C16—H16B	109.5
C1—B5—B4	107.0 (3)	N1—C16—H16C	109.5
C1—B5—Dy^i^	171.5 (2)	H16A—C16—H16C	109.5
B4—B5—Dy^i^	73.34 (18)	H16B—C16—H16C	109.5
C1—B5—H5	126.5	N2—C17—H17A	109.5
B4—B5—H5	126.5	N2—C17—H17B	109.5
Dy^i^—B5—H5	53.8	H17A—C17—H17B	109.5
C2—B6—C1	52.07 (18)	N2—C17—H17C	109.5
C2—B6—H6	122.4 (19)	H17A—C17—H17C	109.5
C1—B6—H6	122 (2)	H17B—C17—H17C	109.5
Si1—C7—H7A	109.5	N2—C18—H18A	109.5
Si1—C7—H7B	109.5	N2—C18—H18B	109.5
H7A—C7—H7B	109.5	H18A—C18—H18B	109.5
Si1—C7—H7C	109.5	N2—C18—H18C	109.5
H7A—C7—H7C	109.5	H18A—C18—H18C	109.5
H7B—C7—H7C	109.5	H18B—C18—H18C	109.5
Si1—C8—H8A	109.5		

Symmetry codes: (i) −*x*+1, −*y*+2, −*z*+1.
